# Pollination Services Provided by Bees in Pumpkin Fields Supplemented with Either *Apis mellifera* or *Bombus impatiens* or Not Supplemented

**DOI:** 10.1371/journal.pone.0069819

**Published:** 2013-07-24

**Authors:** Jessica D. Petersen, Stephen Reiners, Brian A. Nault

**Affiliations:** 1 Department of Entomology, Cornell University, New York State Agricultural Experiment Station, Geneva, New York, United States of America; 2 Department of Horticulture, Cornell University, New York State Agricultural Experiment Station, Geneva, New York, United States of America; University of Northampton, United Kingdom

## Abstract

Pollinators provide an important service in many crops. Managed honey bees (*Apis mellifera* L.) are used to supplement pollination services provided by wild bees with the assumption that they will enhance pollination, fruit set and crop yield beyond the levels provided by the wild bees. Recent declines in managed honey bee populations have stimulated interest in finding alternative managed pollinators to service crops. In the eastern U.S., managed hives of the native common eastern bumble bee (*Bombus impatiens* Cresson) may be an excellent choice. To examine this issue, a comprehensive 2-yr study was conducted to compare fruit yield and bee visits to flowers in pumpkin (*Cucurbita pepo* L.) fields that were either supplemented with *A. mellifera* hives, *B. impatiens* hives or were not supplemented. We compared pumpkin yield, *A. mellifera* flower visitation frequency and *B. impatiens* flower visitation frequency between treatments. Results indicated that supplementing pumpkin fields with either *A. mellifera* or *B. impatiens* hives did not increase their visitation to pumpkin flowers or fruit yield compared with those that were not supplemented. Next, the relationship between frequency of pumpkin flower visitation by the most prominent bee species (*Peponapis pruinosa* (Say), *B. impatiens and A. mellifera*) and fruit yield was determined across all pumpkin fields sampled. Fruit yield increased as the frequency of flower visits by *A. mellifera* and *B. impatiens* increased in 2011 and 2012, respectively. These results suggest that supplementation with managed bees may not improve pumpkin production and that *A. mellifera* and *B. impatiens* are important pollinators of pumpkin in our system.

## Introduction

Bee pollination is an essential ecosystem service for the successful production of many crops [1] and the demand for pollination services is increasing [2]–[6]. The risk of insufficient crop pollination may be increasing as populations of both managed and wild pollinators in North America decline [7]. In areas where bees have declined, crops may not be pollinated sufficiently, leading to yield reduction and potential economic hardships for farmers [8] and a reduced supply of nutritious food [9].

Pumpkin (*Cucurbita pepo* L.) is a monoecious, obligate cross-pollinated crop in the Cucurbitaceae family. To achieve successful pollination and fruit production, insects are required to transfer pollen from male flowers to female flowers [10]. Pumpkin production in North America is concentrated in the Midwest and Northeast regions of the U.S. [11] and cultivars may be grown for consumption (i.e., fruit is processed) or for fall decoration (i.e., jack-o-lanterns). Farmers routinely supplement pumpkin fields with honey bee (*Apis mellifera* L.) hives [12], which is assumed to increase the visitation frequency to pumpkin flowers, and increase fruit yield. However, the effect of supplementation on pumpkin fruit yield has not been empirically examined.

Pollination services provided by *A. mellifera* to U.S. crops were estimated to be worth $14.6 billion [13]. Concerns are mounting that the supply of *A. mellifera* for such services is declining [14], while the demand for pollination services across all pollinator dependent crops is increasing [15]. These concerns have led researchers to identify and assess the pollination efficiency of alternative managed pollinators, including the common eastern bumble bee (*Bombus impatiens* Cresson). On an individual flower-scale, *B. impatiens* is an efficient pollinator of cucurbits in general [16] and pumpkin specifically [17], [18]. In small fields (0.5 ha) of pumpkins supplemented with managed *B. impatiens* hives, fruit set but not fruit weight, was significantly greater than fruit set in fields not supplemented with managed hives [18]. Supplementation with commercially produced *B. impatiens* has been shown to increase fruit yield at the field-scale in blueberry [19]–[21] and raspberry [22] systems. The potential for increasing pumpkin yield by supplementing fields with managed *B. impatiens* hives at the commercial field-scale has not been explored.

Also in light of declining *A. mellifera* populations, researchers are increasingly interested in examining whether wild bee pollinators provide sufficient pollination services to agricultural crops. Evidence suggests that in many cases, wild bees provide substantially more pollination services than previously thought [23], [24]. The contributions of wild bees to crop production have been assessed and confirmed across disparate cropping systems including sunflower [25], coffee [26], sweet cherry [27], blueberry [28], tomato [29], and squash [30], but not yet in pumpkin. In eastern North America, the squash bee, *Peponapis pruinosa* (Say), *B. impatiens* and *A. mellifera* are the most frequently encountered bee species in pumpkin fields and may have the greatest impact on pumpkin production [17], [31].

The first objective of this study was to determine whether supplementing commercial pumpkin fields with either *A. mellifera* or *B. impatiens* hives would increase flower visitation frequency by bees and fruit yield. We hypothesized that fruit yield would be greater in supplemented fields than nonsupplemented ones and that yield would be greatest in fields supplemented with *B. impatiens* because it is a more efficient pollinator than *A. mellifera* in pumpkin [18]. We also hypothesized that *B. impatiens* would visit more pumpkin flowers in fields supplemented with *B. impatiens* compared with nonsupplemented fields, and *A. mellifera* would visit more flowers in fields supplemented with *A. mellifera* than in nonsupplemented ones. The second objective was to determine what factors best predict pumpkin yield, including bee visitation frequency to pumpkin flowers by *B. impatiens*, *A. mellifera* or *P. pruinosa*. Supplementation treatment (i.e., supplementation by *B. impatiens*, *A. mellifera* or no supplementation) and field size were hypothesized to be significant covariates in the model predicting pumpkin yield. Based on the efficiency of *B. impatiens* as a pollinator of individual flowers [18], we hypothesized that an increase in flower visitation frequency by *B. impatiens* would lead to the greatest pumpkin yields.

## Methods

This study was conducted in 23 pumpkin fields in 2011 and 19 fields in 2012 in the Finger Lakes region of New York State, U.S. The fields in 2012 were geographically independent from the fields in 2011. Most fields were in commercial production and ranged in size from 0.5 to 13 hectares. The private land owners of the fields used gave permission to conduct the study. Pumpkin fields of similar size were grouped and then randomly assigned to one of three treatments: *B. impatiens* supplementation (2011: N = 6; 2012: N = 5), *A. mellifera* supplementation (2011: N = 10; 2012: N = 7), or a nonsupplemented control (2011: N = 7; 2012: N = 7).

To compare fruit yield among these treatments, the same jack-o-lantern type variety (*Cucurbita pepo* var. ‘Gladiator’ F1 hybrids) was transplanted into all fields. Although pumpkin varieties planted by the growers in these fields varied, different varieties of pumpkin can cross-pollinate [10]. Gladiator was chosen because it generally produces one large fruit per plant rather than many fruit that would compete for plant resources during development. Transplants were obtained by planting seeds in seedling trays (4×8 cells) containing Cornell soil mix [32] and maintained in the greenhouse. Multiple plantings were made to create a source of 1–2 leaf stage plants that spanned the three-week period growers planted pumpkins in commercial fields. In each pumpkin field, greenhouse-grown plants that matched the size of field-sown plants were transplanted into three plots of 10 plants each (two adjacent rows of five plants; N = 30 transplants per field). Between-row spacing was 2 m and within-row spacing was 1 m. All plots were located at least 20 m from an edge and were arranged to capture the variability of the field topography and edge habitats.

In mid-July, when the plants were just beginning to bloom, *B. impatiens* and *A. mellifera* hives were stocked in pumpkin fields. Commercially reared *B. impatiens* hives were acquired from Koppert Biological Systems, Inc. (Howell, MI). Stocking density was approximately five *B. impatiens* hives per hectare of pumpkins; this stocking density was recommended by Koppert Biological Systems. *B. impatiens* QUADs (boxes of four hives) were placed within the field equidistant from each other. *A. mellifera* hives were supplied by local beekeepers with approximately equal hive strength. Stocking density was approximately one *A. mellifera* hive per hectare of pumpkins; this stocking density was the typical density used by local growers. *A. mellifera* hives were placed along field edges. All fields were separated from each other, and other managed bees (both *A. mellifera* and *B. impatiens*), by at least 1 km. The landscape surrounding each pumpkin field, up to 1 km, was free of *A. mellifera* and *B. impatiens* hives. This is the most common foraging distance observed for *A. mellifera* [33] and *Bombus* spp. [34], [35].

### Data Collection

Bee visits to pumpkin flowers were assessed visually in three transects throughout each field. Transects consisted of two rows of pumpkins, including the area of our small plots, and extended 40 m beyond the plots for a total of 44 m. The total number of bees visiting all pumpkin flowers in each transect was counted once a week for three consecutive weeks (rounds), which spanned the majority of the blooming period. Sampling was conducted between 0600–1100 h (when flowers were open) on sunny to partly cloudy days with minimal wind (<15 km/h). Transects were surveyed for a total of 10 minutes each by slowly walking down the row. Observers scored the number of bee visits to flowers on each plant within the transect, bee species and total number of flowers in the transect. A flower “visit” was recorded if the bee came in contact with the reproductive parts. A subsample of individuals were collected and identified in the lab, and voucher specimens were deposited at the Insect Collections at Cornell University (CUIC), Ithaca, NY. Average bee visitation per flower per field was calculated in the following manner. For each round, bee visits for each species and the number of flowers were summed across the three transects. The total bee visits for each species was divided by the total number of flowers to achieve a flower visitation frequency metric for each round. The visitation frequencies were then averaged across the three sampling rounds for each field and species.

Fruit produced from the transplants in the small plots was harvested and weighed at the end of the growing season. Yield was calculated by averaging the total fruit weight per plant across all three small plots in each field (maximum of N = 30 plants per field). This calculation of yield was most appropriate because (1) maximizing fruit weight is the goal for pumpkin growers and (2) the variety Gladiator typically only produces one fruit per plant, allowing us to better assess the contributions of pollinator visits to flowers on fruit weight. Additionally, the number of viable seeds was counted from two randomly selected fruit per plot in every field (two fruit in each of three plots; N = 6 total fruit per field).

### Statistical Analyses

Viable seed set is a direct measure of pollination success [36], but from the perspective of growers and food supply, fruit weight is a more important variable. A standard least squares mixed model regression was conducted with *plot* nested within *field* as a random factor to test for a relationship between pumpkin weight and the number of viable seeds (i.e., seed set).

One-way analysis of variance (ANOVA) with normal error structure was used to test for differences in fruit yield between *A. mellifera* supplemented fields, *B. impatiens* supplemented fields and nonsupplemented fields. Two one-way ANOVAs were used to analyze the impact of bee supplementation treatment on *A. mellifera* and *B. impatiens* visitation frequency to pumpkin flowers. All effects were considered to be significant at the *P*<0.05 level. Data from both years were combined if the model met the assumptions of ANOVA, otherwise data were analyzed independently for each year.

To determine what factors were influential in predicting pumpkin fruit yield, a candidate set of linear regression models were constructed. Factors in these models included bee visitation frequency to flowers by the most common species (*B. impatiens*, *A. mellifera* and *P. pruinosa*), total bee visitation frequency, whether the field was supplemented with managed bees (*B. impatiens*, *A. mellifera*, or nothing) and field size. Because high fruit yield could be achieved if any one of the three most common bee species was sufficiently abundant, a “total bee visitation frequency” variable was calculated by summing the bee visitation frequencies across all three species for each field. The same set of models was analyzed with average seed set as the dependent variable. A correlation matrix was calculated to test for collinearity among the independent variables included in the full model. Variables with moderately strong correlations (*r*>0.7) were removed from the analysis. Other factors known to impact fruit yield such as disease pressure, fertilization, and between-row spacing [37]–[39] did not vary enough across fields in this study to be considered in the models. Models with both years combined, with and without year included as a variable, produced non-normally distributed studentized residuals, despite attempts to transform variables. Therefore, data in 2011 and 2012 were analyzed independently, which resulted in normally distributed residuals in both years. Model selection analyses were conducted including a supplementation treatment by field size interaction and supplementation by bee visitation frequency interaction, so these terms were eliminated from consideration. Our candidate models included all possible combinations of variables. Akaike Information Criterion, adjusted for small sample size (AICc) was calculated for each model based on likelihood values. We report model weights (w*_i_*) and the relative AICc change (Δ) from the top model (lowest AICc). We calculated relative variable importance weights (calculated as the sum of model weights for all models containing that variable) to determine which variables were the strongest predictors of pumpkin yield. For each variable, we calculated model-averaged parameter estimates and their associated 95% confidence intervals to account for uncertainty in model selection. We also report parameter estimates for the predictors in the top model.

Weather conditions differed between 2011 and 2012 and this could have affected bee population sizes and visitation frequencies. To determine if there was a significant difference in temperature between years, accumulated degree days (base 10°C and starting on January 1) were acquired from seven weather stations in the Finger Lakes region of New York State [40] and post hoc comparisons were made using paired *t*-tests. Mean accumulated degree days across these sites in 2011 were compared with 2012 at four points during the summer when bees are active: May 1, June 1, July 1 and August 1. Artz et al. [17] conducted a study in the same region in 2008 and 2009 and experienced similar climatic differences between years, so the same sets of paired *t*-tests were used to compare accumulated degree days between 2008 and 2009. All statistical analyses described above were performed in R. v. 2.14.2 [41].

## Results

A total of 2390 and 2709 bees were recorded visiting pumpkin flowers in 2011 and 2012, respectively. In both years, there were three dominant species, accounting for 97.7% of the total visits to pumpkin flowers: *P. pruinosa* (2011: N = 1382; 2012: N = 1272), *A. mellifera* (2011: N = 695; 2012: N = 765) and *B. impatiens* (2011: N = 241; 2012: N = 628). There were significantly more *B. impatiens* visiting flowers in 2012 compared with 2011 (t_41_ = 2.72, *P* = 0.01), while *P. pruinosa* and *A. mellifera* visited a similar number of flowers each year (*P*>0.05). The following results focus on the three dominant species as they likely represent the species most responsible for pollination and two are the ones that were supplemented in fields.

### Relationship between Fruit Weight and Viable Seeds

**Figure 1:**
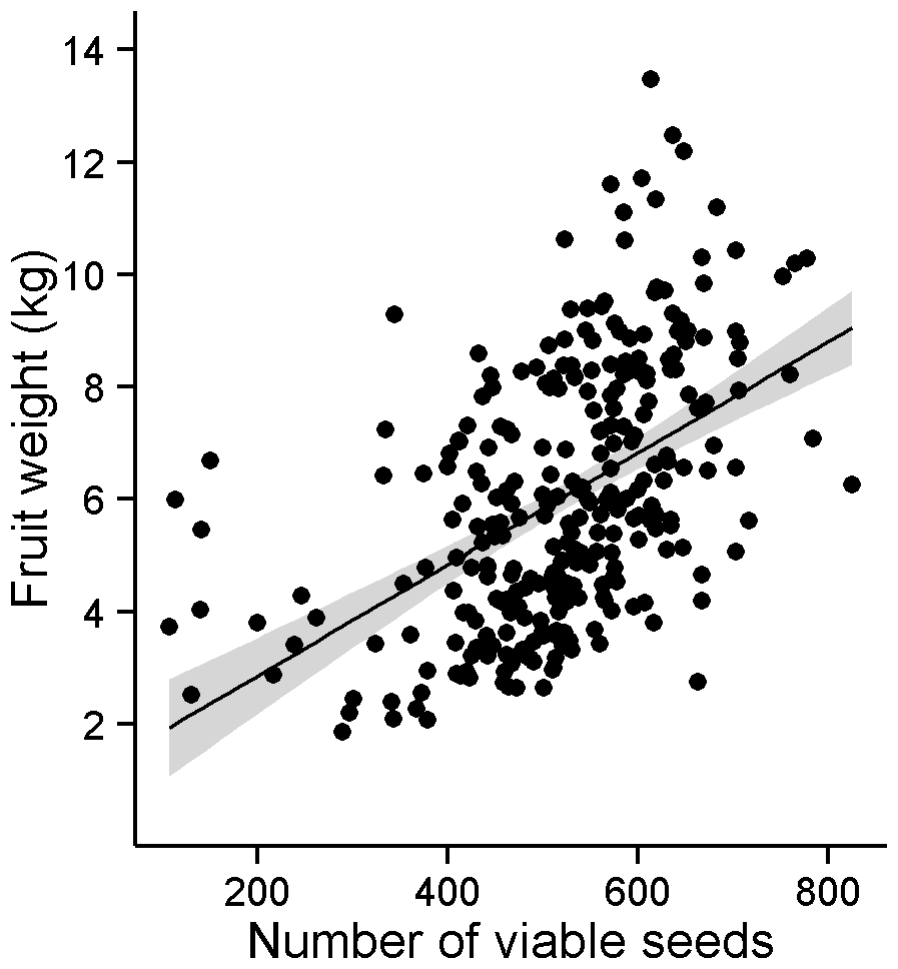
Relationship between fruit weight and viable seeds. Fruit weight was positively correlated with the number of viable seeds (y = 0.86+0.01×, R^2^ = 0.75, *P*<0.001). Gray bands represent 95% confidence limits.

There was a positive relationship between pumpkin fruit weight and the number of viable seeds per pumpkin fruit (*P*<0.0001, R^2^ = 0.75; Fig. 1). The remainder of our analyses focuses on pumpkin fruit yield, calculated as the average of the total fruit weight per plant. We have shown here that average fruit weight indirectly relates to pollination success and is the more important dependent variable from an agricultural perspective. The following analyses were also conducted with average seed set as the dependent variable, but the results did not differ from those presented with yield as the dependent variable.

### Impact of Managed Bee Supplementation on Fruit Yield and Flower Visitation

**Figure 2:**
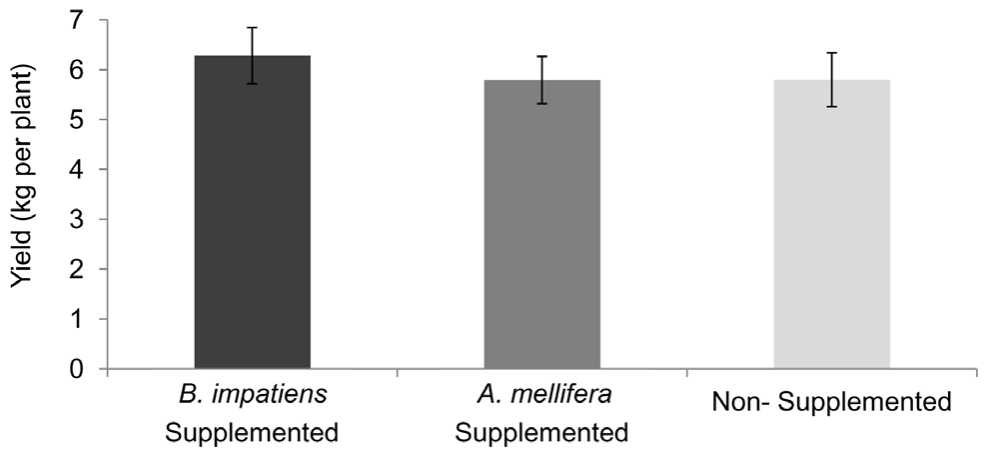
Effects of bee supplementation on fruit yield. Pumpkin fruit yield (average fruit weight per plant ± SEM) was not statistically significantly different among the treatments (*F*_2,39_ = 0.27, *P* = 0.77).

Pumpkin fruit yield in bee-supplemented fields did not differ significantly from yield in nonsupplemented fields (*F*_2,39_ = 0.27, *P* = 0.77) (Fig. 2). The average fruit per plant was 1.11 (SD = 0.18).

Because we failed to reject the null hypothesis (no difference in yield among treatments), we conducted a *post hoc* power analysis to determine if a Type II error occurred that precluded us from detecting a true significant difference in yield among treatments. Using the observed effect size calculated from the treatment means, with α = 0.05, we estimated the power (1-β) using G*Power 3.1.7 [42]. Results indicated that the power of this ANOVA was sufficiently large (1-β = 0.77) to detect a large difference in yield [42].

**Figure 3:**
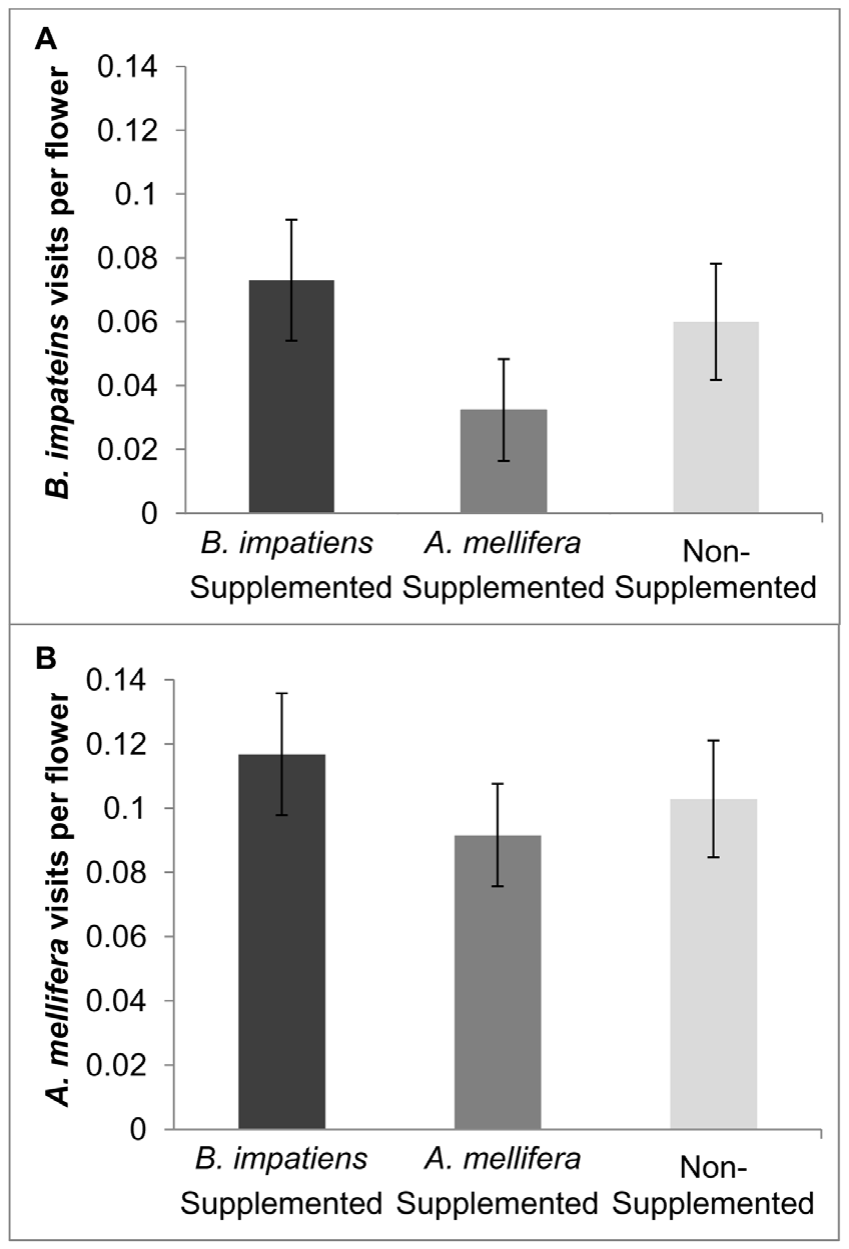
Effects of bee supplementation on flower visitation frequency. Mean (±SEM) *Bombus impatiens* visitation frequency to pumpkin flowers in fields supplemented with *B. impatiens*, or *Apis mellifera* did not differ significantly from visitation frequency in control fields (A). Both years are combined here for simplicity. Mean (±SEM) *A. mellifera* visitation frequency to pumpkin flowers in fields supplemented with *B. impatiens*, or *A. mellifera* did not differ significantly from visitation frequency to flowers in control fields (B).

*B. impatiens* visitation frequency to pumpkin flowers in fields supplemented with *B. impatiens* did not differ significantly from visitation frequency in fields supplemented with *A. mellifera* or control fields in either year of the study (2011: *F*_2,20_ = 1.88, *P* = 0.18; 2012: *F_2,16_* =  = 1.70, *P* = 0.21; Fig. 3A). Similarly, the frequency of *A. mellifera* visits to pumpkin flowers in fields supplemented with *A. mellifera* did not differ significantly from visitation frequency to flowers in fields supplemented with *B. impatiens* or control fields (*F*_2,39_ = 0.28, *P* = 0.76; Fig. 3B).

### Factors Predicting Fruit Yield

**Figure 4:**
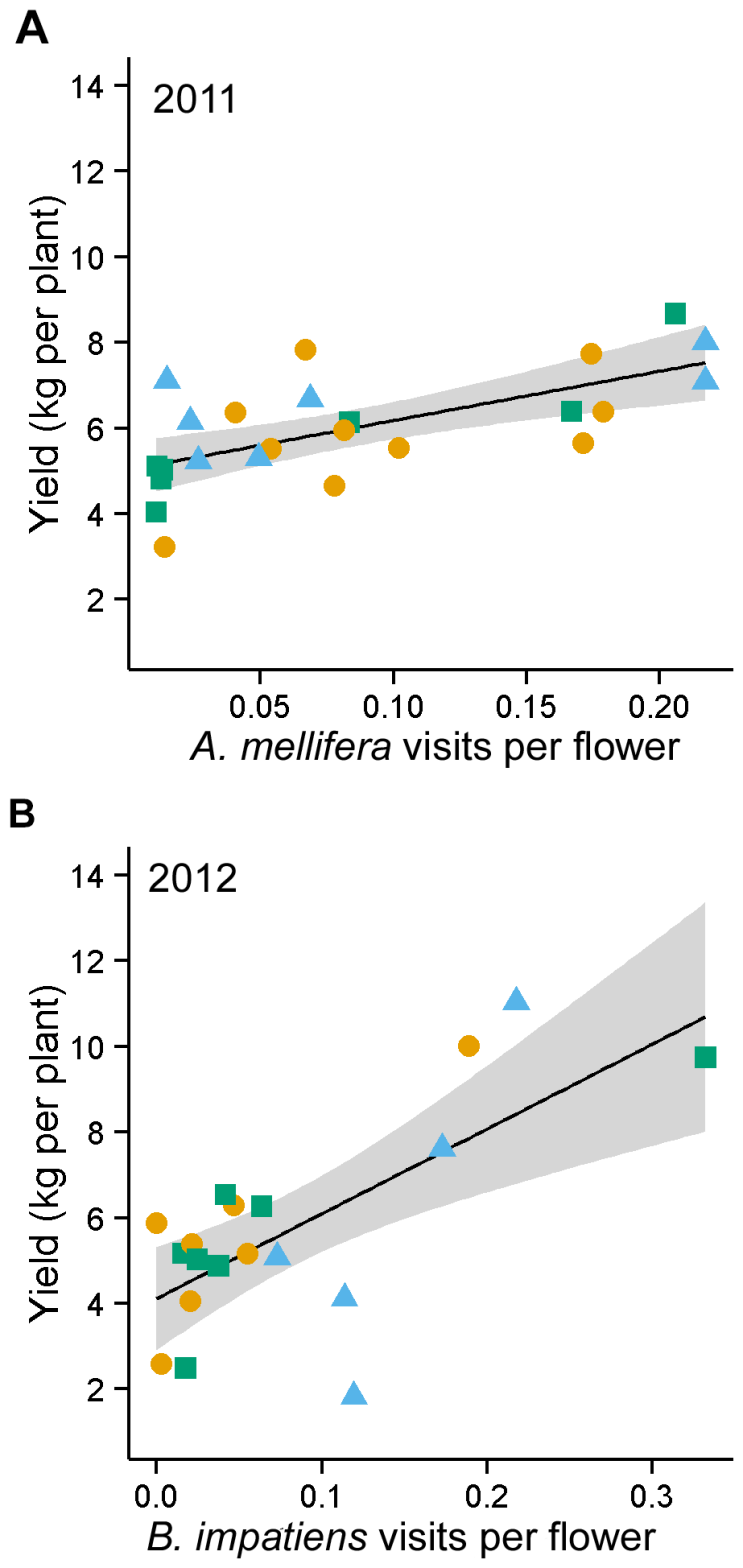
Relationship between yield and bee visitation frequency. Relationship between fruit yield and flower visitation frequency by *Apis mellifera* in 2011 (y = 5.02+11.53×) (A), and *Bombus impatiens* in 2012 (y = 4.11+19.82×) (B). Supplementation treatment (circle = *A. mellifera* supplementation, triangle = *B. impatiens* supplementation, square = nonsupplemented) was illustrated to show the lack of pattern among treatment groups, reinforcing that the point that fruit yield was not influenced by supplementation with managed bees. Supplementation treatment was not included as a factor in these regressions.

Full models that included visitation frequency for *A. mellifera*, *B. impatiens*, and *P. pruinosa*, supplementation treatment and field size significantly predicted fruit yield (2011: *P = *0.04; 2012: *P* = 0.05). Only two variables were highly correlated in both years: *P. pruinosa* visitation frequency and total bee visitation frequency (Table S1 and S2). Total bee visitation frequency was not included in the model selection. In 2011, model selection and model average results indicated that *A. mellifera* visits per flower was the most important predictor of pumpkin yield (Table 1). The top model included both *A. mellifera* and *P. pruinosa* visits per flower, but only *A. mellifera* had a significant and positive association with pumpkin yield (Fig. 4A). In addition to the top model, there was one competing model including *A. mellifera* visits per flower (Table 2). In 2011, *P. pruinosa* visits per flower was marginally important in fitting the model, but was not a significant predictor of yield (Table 1). In 2012, model selection indicated that *B. impatiens* visits per flower was the most important predictor of pumpkin yield (Table 3) and was positively correlated with pumpkin yield (Fig. 4B). There were no competing models in 2012, and the next best models had low model weights (Table 4). Overall, only *A. mellifera* and *B. impatiens* visits per flower were important predictors of pumpkin yield in 2011 and 2012, respectively. Other covariates in the full model including supplementation treatment and field size were poor predictors of pumpkin yield in this 2-yr study.

**Table 1:** Model selection results for 2011.

Variable	Relative variable weight	Estimate from top model (lower/upper CI)	Estimate from model average (lower/upper CI)
*A. mellifera*	1.0	29.56 (15.84/43.28)*	27.79 (14.21/41.38)*
*P. pruinosa*	0.64	2.78 (−0.11/5.67)	2.72 (−0.08/5.51)
*B. impatiens*	0.15	–	−4.82 (−49.17/39.52)
Field size	0.14	–	0.0003 (−0.11/0.11)
Treatment	0.03	–	*A. mellifera* supplemented: −0.28 (−2.59/2.04)
			*B. impatiens* supplemented: 1.28 (−1.12/3.77)

Relative variable importance weights and parameter estimates with 95% confidence intervals for all variables from models predicting pumpkin yield in 2011. Significant factors are denoted with * (*P*<0.05). Variables included: bee visitation frequency to pumpkin flowers (*Apis mellifera*, *Peponapis pruinosa*, and *Bombus impatiens*), field size, and supplementation treatment (*B. impatiens* supplemented, *A. mellifera* supplemented and nonsupplemented).

**Table 2:** AICc model selection results for 2011 for models that fell within 4 AICc of the top model.

Model	K	ΔAICc	Model weight (w*_i_*)
*P. pruinosa, A. mellifera*	4	0	0.45
*A. mellifera*	3	1.46	0.22
*P. pruinosa, A. mellifera*, Field size	5	3.22	0.09
*P. pruinosa, A. mellifera, B. impatiens*	5	3.22	0.09

Independent variables included in the model selection to predict pumpkin yield included bee visitation frequency to pumpkin flowers (*Bombus impatiens*, *Apis mellifera* and *Peponapis pruinosa*), supplementation treatment (*B. impatiens* supplemented, *A. mellifera* supplemented and nonsupplemented) and field size.

**Table 3:** Model selection results for 2012.

Variable	Relative variable weight	Estimate from top model (lower/upper CI)	Estimate from model average (lower/upper CI)
*A. mellifera*	0.17	–	−5.93 (−26.83/14.98)
*P. pruinosa*	0.12	–	−0.03 (−10.52/10.46)
*B. impatiens*	1.0	43.70 (21.34/66.06)*	45.08 (21.22/68.94)*
Field size	0.15	–	0.086 (−0.21/0.39)
Treatment	0.07	–	*A. mellifera* supplemented: 1.23 (−3.28/5.75)
			*B. impatiens* supplemented: −2.78 (−7.90/2.34)

Relative variable importance weights and parameter estimates with 95% confidence intervals for all variables from models predicting pumpkin yield in 2012. Significant factors are denoted with an asterisk (*) (*P*<0.05). Variables included: bee visitation frequency to pumpkin flowers (*Apis mellifera*, *Peponapis pruinosa*, and *Bombus impatiens*), field size, and supplementation treatment (*B. impatiens* supplemented, *A. mellifera* supplemented and nonsupplemented).

**Table 4:** AICc model selection results for 2012 for models that fell within 4 AICc of the top model.

Model	K	ΔAICc	Model weight (w*_i_*)
*B. impatiens*	3	0	0.53
*B. impatiens*, Field size	4	2.80	0.13
*B. impatiens, A. mellifera*	4	2.82	0.13
*B. impatiens, P. pruinosa*	4	3.26	0.10

Independent variables included in the model selection to predict pumpkin yield included bee visitation frequency to pumpkin flowers (*Bombus impatiens*, *Apis mellifera* and *Peponapis pruinosa*), supplementation treatment (*B. impatiens* supplemented, *A. mellifera* supplemented and nonsupplemented) and field size.

### Association between B. Impatiens Visitation to Flowers and Seasonal Temperatures

**Figure 5:**
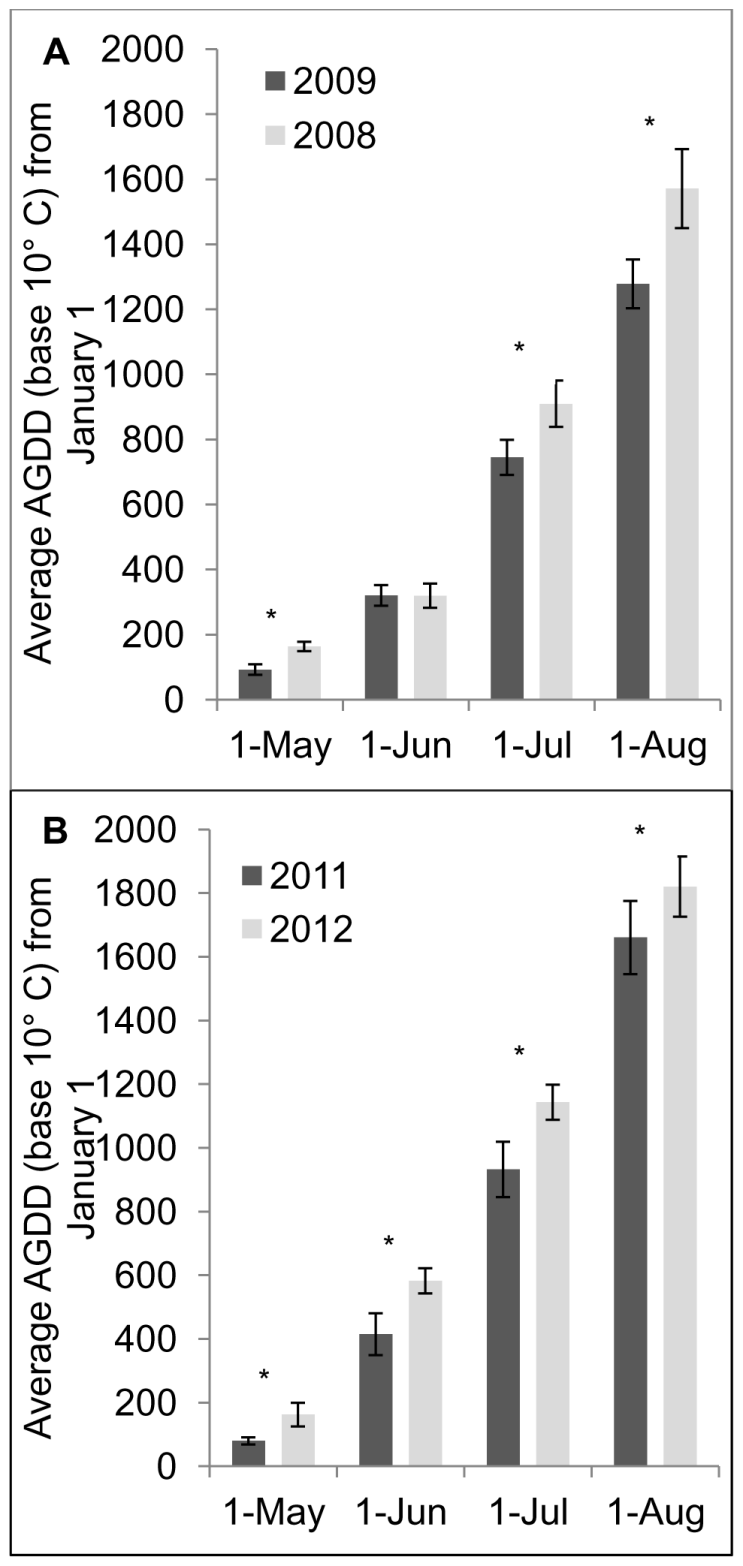
*B.* *impatiens* visits to pumpkin flowers increases in warmer years. Average accumulated growing degree days (AGGD), base 10°C from seven weather stations, starting January 1 through May 1, June 1, July 1 and August 1 for 2008 and 2009 (A), and 2011 and 2012 (B). Significant paired *t*-test results are indicated with an asterisk (*) above the column (*P*<0.05). In all significant pair-wise comparisons, 2008 and 2012, where *B. impatiens* visits to pumpkin flowers were greatest, were warmer than 2009 and 2011 respectively.

Accumulated growing degree days were significantly greater from May through August in 2008 than in 2009 and significantly greater in 2012 than in 2011 (Fig. 5). The only exception was that differences were not evident between growing degree days in June 2008 and 2009. There were significantly more *B. impatiens* visits to pumpkin flowers in 2008 (the warmer year) compared with 2009 [17]. The same trend held true for the current study in which there were significantly more *B. impatiens* visits to pumpkin flowers in 2012 (the warmer year) compared with 2011. Although our sample size is low (N = 2) there was an obvious trend towards greater visitation frequency by *B. impatiens* in warmer years.

## Discussion

Contrary to predictions, supplementing pumpkin fields with managed pollinators did not increase pollinator visitation to flowers nor did it improve fruit yields. Yet, our expectation that pumpkin yield would be positively correlated with the frequency of *B. impatiens* visits to pumpkin flowers was confirmed, but only in 2012, which was the warmest of the two years and the year that *B. impatiens* visits to pumpkin flowers was greatest. *A. mellifera* also was confirmed as an important pollinator of pumpkin, but only in 2011, as fruit yield significantly increased as its visitation frequency to flowers increased. Pumpkin fruit yield in control fields was high and similar to those in fields supplemented with managed bees, indicating that wild *B. impatiens* and feral or nearby managed *A. mellifera* are playing important roles in pumpkin pollination and commercial production in our study system.

Previous research has shown an increase in pumpkin yield in fields supplemented with *A. mellifera* compared with nonsupplemented fields [43]. However, the treatments in that study were not replicated and the experiment was conducted in small plots consisting of 10 plants. Our results are consistent with those from other studies that supplemented crops with *A. mellifera* and failed to generate greater *A. mellifera* visitation frequency to pumpkin flowers compared with nonsupplemented fields [44], [45] but see [17]. Our study was the first to assess the effects of *B. impatiens* supplementation on pumpkin yield and bee visits to flowers. Why did supplementation not have an effect on pumpkin yield or bee visitation frequency for either *A. mellifera* or *B. impatiens*? We suggest three possible explanations for these results below.

Stocking density of both *A. mellifera* and *B. impatiens* hives could have been too low to detect an increase in flower visitation frequency and subsequently fruit yield. Research in blueberries has shown that *B. impatiens* is a near-nest central place forager [20] increasing yield up to 150 m from the hive. *Bombus* spp. exhibit flower visit constancy, even when faced with changes in resource availability [46]. Given these behaviors, one might expect *B. impatiens* to be a good candidate for supplementation at the appropriate stocking density. The effects of different stocking densities of *B. impatiens* hives in pumpkin fields have not been empirically tested like they have been in blueberry [19]. There is a wide range in recommended *A. mellifera* stocking densities for pumpkin, but the average density (3.8 hives per ha) reported from a literature review published in 2000 was considerably higher than the density used in this study (1 hive per ha), which is the stocking density most commonly used in our region of New York [10].

The minimum distance of 1 km from managed *A. mellifera* hives might not have been far enough apart to sufficiently evaluate differences in visitation frequency between fields supplemented with *A. mellifera* and nonsupplemented fields. Average foraging distances by *A. mellifera* vary by region, time and resource availability [47], [48]. Foraging distance estimation during a mass blooming flower (*Calluna vulgaris* L.) indicated that 50% of individuals foraged more than 6 km [49]. Thus, *A. mellifera* has the potential for regular, long-distance foraging flights for certain resources, but measuring the distance a bee will travel for pumpkin nectar or pollen has not been investigated.

Managed bees placed in pumpkin fields might forage outside of these fields either because the field is already saturated with bees, or they desire alternative resources, or both. If pumpkin fields are typically saturated with wild pollinators during bloom, supplementing fields with managed bees likely will not enhance flower visitation frequency. This saturation effect has been shown at various spatial scales in a coffee system [50]. When framed in a yield context, pollen may not be a limiting factor in producing the maximum yield in pumpkin fields in this region. In other words, wild bees and nearby managed or feral *A. mellifera* may be providing the maximum pollination services possible. Field experiments assessing pollen limitation explicitly could shed light on the results found in the current study.

Managed bees might leave pumpkin fields because pumpkin pollen or nectar may be less desirable than other resources blooming concurrently. Previous research suggests that cucurbits, including pumpkin are less rewarding than other competing flowering resources [10]. *A. mellifera* can detect and evaluate the sugar concentration of nectar from plants and will choose sweeter nectar [51]. The sugar concentration of pumpkin nectar (35–50%) [52–52] is within the preferential range of *A. mellifera* [53]. *Cucurbita* pollen is rich in crude protein (38.6%), but previous research suggests that more nutritious pollen is not necessary preferred by bees [54]. The degree of pumpkin pollen or nectar foraging fidelity of either *A. mellifera* or *B. impatiens* has not yet been explored.

Our results support previous research that suggests *B. impatiens* is an important pollinator of cucurbits [55], [56]. The greatest yield was produced in 2012 when *B. impatiens* visitation rate was the highest (i.e., the peak yield was greater in Fig. 4B compared with Fig. 4A). The degree to which wild bees provide sufficient pollination services may depend on various factors such as weather, landscape heterogeneity and alternative bloom context [57]. Bumble bee colony growth rate depends primarily on pollen availability [58]. Thus, a warm spring would promote earlier plant development and pollen availability that could provide *B. impatiens* to colonies an opportunity to increase more quickly and reach high population sizes during the period pumpkins are blooming. We speculate that this was the case in 2012. Our data from non-supplemented fields also suggest that there is variability in the number of visits by wild and feral bees to pumpkin flowers among fields. Factors such as farming practices (e.g., tillage, organic farming) and the landscape surrounding fields may influence such variability [44], [56], [59]. The surrounding landscape in this region is heterogeneous and the matrix surrounding the pumpkin fields used in this study varied in terms of diversity and available complementary resources. The influence of these abiotic factors on bee visitation frequency has not been examined in pumpkin systems and may be important for growers in determining when and where to plant their fields to benefit from pollination services by wild bees.

The wild bee, *P. pruinosa* was not an important predictor of pumpkin yield in either year of our study. Although it is a cucurbit specialist, another study from the same region demonstrated the relative inefficiency of *P. pruinosa* as a pollinator of pumpkin on an individual flower basis [18], but ours was the first study to connect visits to flowers with yield. Field tillage can have a profound effect on *P. pruinosa* density in pumpkin fields [44]. Although all of the fields in our study were tilled, most of the visits to flowers were made by *P. pruinosa*. If this species played an important role in yield, we would have likely seen an effect given the large between-field variability in visitation frequency (Tables S1 and S2). Because total bee visitation frequency was correlated with *P. pruinosa*, and this variable was not a good predictor of pumpkin fruit yield, we can conclude that this species was not important as a pollinator of pumpkin.

Our results suggest that supplementing pumpkin fields with either *A. mellifera* or *B. impatiens* at the stocking densities presented here may not be profitable to growers. Future research should focus the local and landscape factors that may help identify when and where wild pollinator populations may be low and could benefit by supplementation.

## Supporting Information

Table S1 **Correlation matrix (** ***r*** **) and summary statistics for 2011 pumpkin flower visitation frequencies for each bee species, all species combined, and field size data.** Significant relationships between variables (*: *P*<0.05, **P<0.01) are indicated.
(DOCX)

Table S2 **Correlation matrix (** ***r*** **) and summary statistics for 2012 pumpkin flower visitation frequencies for each bee species, all species combined, and field size data.** Significant relationships between variables (*: *P*<0.05, **P<0.01) are indicated.
(DOCX)
