# Supplementary material for: Pollination Services Provided by Bees in Pumpkin Fields Supplemented with Either Apis mellifera or Bombus impatiens or Not Supplemented
Source: PLoS One. 2013 Jul 24;8(7):e69819. doi: 10.1371/journal.pone.0069819 (PMC3722171; doi:10.1371/journal.pone.0069819)
Supplement: Table S1 — Correlation matrix ( r ) and summary statistics for 2011 pumpkin flower visitation frequencies for each bee species, all species combined, and field size data. Significant relationships between variables (*: P<0.05, **P<0.01) are indicated. (DOCX) [file pone.0069819.s001.docx]

Table S1.

|  | *P. pruinosa* | *B. impatiens* | *A. mellifera* | Total bee | Field size (ha) |
| --- | --- | --- | --- | --- | --- |
| *B. impatiens* | -0.16 | -- | -- | -- | -- |
| *A. mellifera* | -0.33 | 0.05 | -- | -- | -- |
| Total bee | 0.98** | -0.09 | -0.12 | -- |  |
| Field size | -0.11 | -0.39 | -0.13 | -0.17 | -- |
| Mean | 0.20 | 0.03 | 0.09 | 0.32 | 5.12 |
| SD | 0.35 | 0.02 | 0.07 | 0.33 | 3.49 |
| Min | 0.01 | 0.003 | 0.01 | 0.10 | 0.49 |
| Max | 1.54 | 0.08 | 0.22 | 1.58 | 13.18 |
