# Supplementary material for: Pollination Services Provided by Bees in Pumpkin Fields Supplemented with Either Apis mellifera or Bombus impatiens or Not Supplemented
Source: PLoS One. 2013 Jul 24;8(7):e69819. doi: 10.1371/journal.pone.0069819 (PMC3722171; doi:10.1371/journal.pone.0069819)
Supplement: Table S2 — Correlation matrix ( r ) and summary statistics for 2012 pumpkin flower visitation frequencies for each bee species, all species combined, and field size data. Significant relationships between variables (*: P<0.05, **P<0.01) are indicated. (DOCX) [file pone.0069819.s002.docx]

Table S2.

|  | *P. pruinosa* | *B. impatiens* | *A. mellifera* | Total bee | Field size (ha) |
| --- | --- | --- | --- | --- | --- |
| *B. impatiens* | -0.18 | -- | -- | -- | -- |
| *A. mellifera* | -0.41 | 0.26 | -- | -- | -- |
| Total bee | 0.72** | 0.39 | 0.21 | -- | -- |
| Field size | -0.09 | -0.47* | -0.22 | -0.48 | -- |
| Mean | 0.17 | 0.08 | 0.12 | 0.37 | 4.62 |
| SD | 0.20 | 0.09 | 0.10 | 0.20 | 3.11 |
| Min | 0.01 | 0 | 0.01 | 0.13 | 0.43 |
| Max | 0.85 | 0.33 | 0.42 | 0.96 | 12.36 |
